# Aqua­[2-(2-pyrid­yl)-1,8-naphthyridine-κ^2^ *N* ^1^,*N* ^2^](2,2′:6′,2′′-terpyridine-κ^3^ *N*,*N*′,*N*′′)ruthenium(II) bis­(hexa­fluorido­phosphate) acetone sesquisolvate

**DOI:** 10.1107/S1600536811016485

**Published:** 2011-05-11

**Authors:** Dai Oyama, Kazumi Yuzuriya, Tsugiko Takase

**Affiliations:** a Department of Industrial Systems Engineering, Cluster of Science and Technology, Fukushima University, 1 Kanayagawa, Fukushima 960-1296, Japan; b Department of Environmental Systems Management, Cluster of Science and Technology, Fukushima University, 1 Kanayagawa, Fukushima 960-1296, Japan; c Center for Practical and Project-Based Learning, Cluster of Science and Technology, Fukushima University, 1 Kanayagawa, Fukushima 960-1296, Japan

## Abstract

The asymmetric unit of the title compound, [Ru(C_13_H_9_N_3_)(C_15_H_11_N_3_)(H_2_O)](PF_6_)_2_·1.5C_3_H_6_O, consists of two crystallographically independent Ru^II^ complexes. Each complex is approximately octa­hedral with the Ru^II^ atom bound by an *N*,*N*′-coordinated 2-(2-pyrid­yl)-1,8-naphthyridine (pynp) ligand, a meridional 2,2′:6′,2′′-terpyridine (tpy) ligand and one aqua ligand. The tpy ligand is coordinated in a planar tridentate fashion with the central N atom closest to the Ru^II^ atom. The aqua ligand is *trans* to the pyridine N atom of pynp. The long Ru—O distances [2.150 (5) and 2.138 (5) Å] are typical for aqua ligands in polypyridyl ruthenium complexes. In the crystal, both intra­molecular O—H⋯N and inter­molecular O—H⋯O hydrogen bonds are observed.

## Related literature

For synthetic details, see: Campos-Fernandez *et al.* (2002 ▶); Tseng *et al.* (2008 ▶). For related structures, see: Zong *et al.* (2004 ▶); Tomon *et al.* (2005 ▶); Yang *et al.* (2005 ▶); Qvortrup *et al.* (2007 ▶). For the redox properties of the pynp ligand, see: Oyama *et al.* (2011 ▶). For general background to catalytic water oxidation using mononuclear ruthenium complexes, see: Tseng *et al.* (2008 ▶); Masaoka & Sakai (2009 ▶); Yoshida *et al.* (2010 ▶).
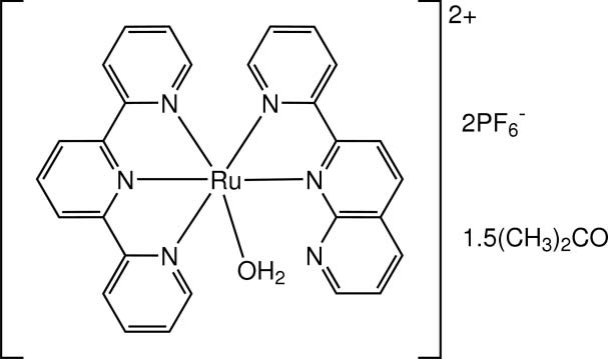


## Experimental

### 

#### Crystal data

[Ru(C_13_H_9_N_3_)(C_15_H_11_N_3_)(H_2_O)](PF_6_)_2_·1.5C_3_H_6_O
*M* *_r_* = 936.64
Triclinic, 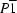

*a* = 8.5768 (10) Å
*b* = 19.3495 (17) Å
*c* = 23.537 (3) Å
α = 69.814 (4)°
β = 89.511 (5)°
γ = 78.550 (5)°
*V* = 3585.6 (7) Å^3^
*Z* = 4
Mo *K*α radiation
μ = 0.63 mm^−1^
*T* = 93 K
0.130 × 0.080 × 0.004 mm

#### Data collection

Rigaku VariMax diffractometer with Saturn CCD
Absorption correction: multi-scan (*REQAB*; Rigaku, 1998 ▶) *T*_min_ = 0.881, *T*_max_ = 0.997
32576 measured reflections
12546 independent reflections
9436 reflections with *I* > 2σ(*I*)
*R*_int_ = 0.033

#### Refinement

*R*[*F* ^2^ > 2σ(*F* ^2^)] = 0.054
*wR*(*F* ^2^) = 0.152
*S* = 1.05
12546 reflections
1027 parameters
11 restraints
H atoms treated by a mixture of independent and constrained refinement
Δρ_max_ = 1.74 e Å^−3^
Δρ_min_ = −1.21 e Å^−3^

### 

Data collection: *CrystalClear-SM Expert* (Rigaku, 2009 ▶); cell refinement: *CrystalClear-SM Expert*; data reduction: *CrystalClear-SM Expert*; program(s) used to solve structure: *SIR92* (Altomare *et al.*, 1994 ▶); program(s) used to refine structure: *SHELXL97* (Sheldrick, 2008 ▶); molecular graphics: *ORTEP-3* (Farrugia, 1997 ▶); software used to prepare material for publication: *CrystalStructure* (Rigaku, 2010 ▶).

## Supplementary Material

Crystal structure: contains datablocks global, I. DOI: 10.1107/S1600536811016485/is2707sup1.cif

Structure factors: contains datablocks I. DOI: 10.1107/S1600536811016485/is2707Isup2.hkl

Additional supplementary materials:  crystallographic information; 3D view; checkCIF report

## Figures and Tables

**Table 1:** Selected bond lengths (Å)

Ru1—O1	2.150 (5)
Ru1—N1	2.073 (4)
Ru1—N2	1.956 (4)
Ru1—N3	2.076 (4)
Ru1—N4	2.019 (5)
Ru1—N5	2.098 (4)
Ru2—O2	2.138 (5)
Ru2—N7	2.066 (4)
Ru2—N8	1.952 (4)
Ru2—N9	2.072 (4)
Ru2—N10	2.023 (6)
Ru2—N11	2.097 (4)

**Table 2:** Hydrogen-bond geometry (Å, °)

*D*—H⋯*A*	*D*—H	H⋯*A*	*D*⋯*A*	*D*—H⋯*A*
O1—H1*A*⋯N6	0.81 (3)	1.91 (3)	2.647 (5)	151 (4)
O1—H1*B*⋯O4	0.81 (4)	1.98 (4)	2.773 (6)	167 (5)
O2—H2*A*⋯N12	0.80 (3)	1.91 (3)	2.637 (5)	151 (4)
O2—H2*B*⋯O5	0.81 (4)	1.98 (4)	2.762 (6)	161 (4)

**Fig. 1.**
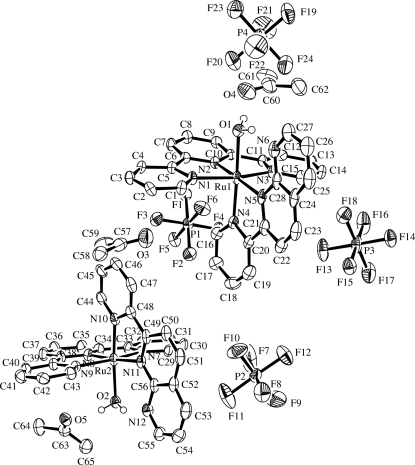
The molecular structure of the title compound, with atom labels and 50% probability displacement ellipsoids. H atoms (except aqua ligand) have been omitted for clarity.

## supplementary crystallographic information

### Comment

Polypyridine complexes of ruthenium(II) have been extensively studied as water oxidation catalysts. In particular, mononuclear complexes containing both tridentate and bidentate pyridyl ligands show good catalytic activity (Tseng *et al.*, 2008; Masaoka & Sakai, 2009; Yoshida *et al.*, 2010). On the other hand, the bidentate naphthyridine, 2-(2-pyridyl)-1,8-naphthyridine (pynp), is a useful ligand for mononuclear systems, which undergoes a ligand-based redox reaction (Oyama *et al.*, 2011). For complexes with the pynp ligand, there is a stereochemical problem with regard to binding of the pynp ligand because it is unsymmetrical structure. For example, only one isomer is isolated and its structure has been determined by X-ray crystallography in the case of the [Ru(tpy)(pynp)Cl]^+^ (tpy = 2,2':6',2''- terpyridine) complex (Tseng *et al.*, 2008). In the present work, [Ru(tpy)(pynp)(H_2_O)](PF_6_)_2_.1.5(CH_3_)_2_CO was newly prepared to investigate its detailed structure.

The crystal structure of the title compound contains two independent [Ru(tpy)(pynp)(H_2_O)]^2+^ complex cations in the asymmetric unit. The ligand environment about the Ru^II^ atom is distorted octahedral, with the terpyridyl ligand coordinated in a meridional fashion, the pynp ligand coordinated in a *cis* fashion and the aqua ligand *trans* to the pyridine N atom of pynp (Fig. 1): the naphthyridine moiety of the pynp ligand is directed toward the proximal aqua ligand. This geometry is different from that of the corresponding chlorido complex: the Cl atom is located *trans* to the naphthyridine nitrogen atom of pynp (Tseng *et al.*, 2008). The Ru—O [2.150 (5) and 2.138 (5) Å] and Ru—N [1.952 (4)–2.098 (4) Å] are in the expected ranges (Table 1) (Yang *et al.*, 2005; Qvortrup *et al.*, 2007; Tseng *et al.*, 2008). The acetone solvate molecules form O···H—O hydrogen bonds to the aqua ligand (Table 2), with D···A distances of 2.773 (6) Å (O1···O4) and 2.762 (6) Å (O2···O5). In addition, the short O···N distances between the aqua ligand and the non-coordinating pynp nitrogen [2.647 (5), 2.637 (5) Å] and the large O—H···N angles [151 (4)°] suggest intramolecular hydrogen bonds between these ligands (Table 2) (Zong *et al.*, 2004; Tomon *et al.*, 2005). The synergistic effect of these hydrogen bonds helps to stabilize the structure.

### Experimental

The ligand 2-(2-pyridyl)-1,8-naphthyridine (pynp) was prepared and purified as described by Campos-Fernandez *et al.* (2002). The precursor, [Ru(tpy)(pynp)Cl]Cl, was prepared according to known procedure (Tseng *et al.*, 2008). Visible light (λ > 400 nm) irradiation of two isomeric mixtures of [Ru(tpy)(pynp)Cl]^+^ in acetone/water solution leads the formation of only one isomer of [Ru(tpy)(pynp)(H_2_O)]^2+^. X-ray quality crystals were grown by the diffusion of diethyl ether into an acetone solution of the complex over a few days.

### Refinement

Phenyl H atoms were fixed at C—H distances of 0.95 Å and refined as riding, with *U*_iso_(H) = 1.2*U*_eq_(C). Methyl H atoms were placed with idealized threefold symmetry and fixed C—H distances of 0.98 Å, and they were refined in a riding model, with *U*_iso_(H) = 1.5*U*_eq_(C). H atoms bonded to O atoms were located in a difference Fourier map and were restrained to be equal with the *SADI* command in *SHELXL97* (Sheldrick, 2008). The isotropic displacement parameters *U*_eq_(H) were set at 1.2*U*_eq_(O). The highest residual electron density peak and the deepest hole are located 0.87 and 0.81 Å, respectively, from atom Ru1.

### Crystal data

**Table:**

[Ru(C_13_H_9_N_3_)(C_15_H_11_N_3_)(H_2_O)](PF_6_)_2_·1.5C_3_H_6_O	*Z* = 4
*M**_r_* = 936.64	*F*(000) = 1880.00
Triclinic, *P*1	*D*_x_ = 1.735 Mg m^−^^3^
Hall symbol: -P 1	Mo *K*α radiation, λ = 0.71075 Å
*a* = 8.5768 (10) Å	Cell parameters from 8149 reflections
*b* = 19.3495 (17) Å	θ = 3.1–25.0°
*c* = 23.537 (3) Å	µ = 0.63 mm^−^^1^
α = 69.814 (4)°	*T* = 93 K
β = 89.511 (5)°	Platelet, red
γ = 78.550 (5)°	0.13 × 0.08 × 0.004 mm
*V* = 3585.6 (7) Å^3^	

### Data collection

**Table:**

Rigaku VariMax diffractometer with Saturn CCD	9436 reflections with *F*^2^ > 2σ(*F*^2^)
Detector resolution: 7.111 pixels mm^-1^	*R*_int_ = 0.033
ω scans	θ_max_ = 25.0°
Absorption correction: multi-scan (*REQAB*; Rigaku, 1998)	*h* = −10→10
*T*_min_ = 0.881, *T*_max_ = 0.997	*k* = −23→22
32576 measured reflections	*l* = −28→22
12546 independent reflections	

### Refinement

**Table:**

Refinement on *F*^2^	Secondary atom site location: difference Fourier map
*R*[*F*^2^ > 2σ(*F*^2^)] = 0.054	Hydrogen site location: inferred from neighbouring sites
*wR*(*F*^2^) = 0.152	H atoms treated by a mixture of independent and constrained refinement
*S* = 1.05	*w* = 1/[σ^2^(*F*_o_^2^) + (0.0773*P*)^2^ + 8.7987*P*] where *P* = (*F*_o_^2^ + 2*F*_c_^2^)/3
12546 reflections	(Δ/σ)_max_ = 0.001
1027 parameters	Δρ_max_ = 1.74 e Å^−^^3^
11 restraints	Δρ_min_ = −1.21 e Å^−^^3^
Primary atom site location: structure-invariant direct methods	

### Special details

**Table:**

Geometry. ENTER SPECIAL DETAILS OF THE MOLECULAR GEOMETRY
Refinement. Refinement was performed using all reflections. The weighted *R*-factor (*wR*) and goodness of fit (*S*) are based on *F*^2^. *R*-factor (gt) are based on *F*. The threshold expression of *F*^2^ > 2.0 σ(*F*^2^) is used only for calculating *R*-factor (gt).

### Fractional atomic coordinates and isotropic or equivalent isotropic displacement parameters (Å^2^)

**Table:**

	*x*	*y*	*z*	*U*_iso_*/*U*_eq_	
Ru1	0.50963 (4)	0.23909 (2)	0.271129 (17)	0.02292 (12)	
Ru2	0.78561 (5)	0.26832 (2)	0.795674 (18)	0.02785 (12)	
P1	0.83061 (15)	0.10593 (7)	0.53713 (6)	0.0277 (3)	
P2	0.75319 (17)	0.59591 (8)	0.38813 (7)	0.0365 (4)	
P3	0.92511 (16)	0.45118 (8)	0.13413 (6)	0.0362 (4)	
P4	0.29409 (18)	0.08968 (8)	0.08243 (7)	0.0405 (4)	
F1	0.8731 (4)	0.01852 (15)	0.54605 (13)	0.0345 (7)	
F2	0.7878 (4)	0.19352 (16)	0.52916 (14)	0.0396 (8)	
F3	0.6555 (4)	0.09870 (17)	0.55914 (15)	0.0405 (8)	
F4	1.0064 (4)	0.11374 (17)	0.51654 (14)	0.0399 (8)	
F5	0.8949 (4)	0.08587 (16)	0.60583 (13)	0.0363 (7)	
F6	0.7705 (4)	0.12657 (18)	0.46860 (14)	0.0468 (8)	
F7	0.8444 (6)	0.5167 (3)	0.4301 (2)	0.1006 (19)	
F8	0.6561 (5)	0.6758 (2)	0.34368 (17)	0.0598 (10)	
F9	0.8949 (5)	0.6347 (3)	0.3935 (3)	0.0795 (14)	
F10	0.6071 (5)	0.5629 (3)	0.3744 (3)	0.0861 (16)	
F11	0.6716 (5)	0.6149 (3)	0.44271 (17)	0.0784 (14)	
F12	0.8355 (5)	0.5798 (3)	0.33183 (18)	0.0640 (11)	
F13	0.8694 (4)	0.4203 (2)	0.20169 (14)	0.0542 (10)	
F14	0.9796 (4)	0.48126 (17)	0.06697 (13)	0.0426 (8)	
F15	0.7974 (4)	0.52757 (17)	0.12089 (14)	0.0432 (8)	
F16	1.0529 (4)	0.3745 (2)	0.14758 (16)	0.0579 (11)	
F17	1.0552 (5)	0.4861 (3)	0.15705 (16)	0.0623 (11)	
F18	0.7942 (4)	0.41522 (18)	0.11189 (16)	0.0473 (9)	
F19	0.3305 (5)	0.0828 (3)	0.01800 (16)	0.0624 (11)	
F20	0.2550 (6)	0.0970 (3)	0.14594 (19)	0.0736 (12)	
F21	0.4306 (6)	0.0186 (3)	0.1137 (2)	0.0815 (14)	
F22	0.1554 (5)	0.1601 (3)	0.0505 (3)	0.0813 (14)	
F23	0.1686 (5)	0.0362 (2)	0.09049 (17)	0.0627 (11)	
F24	0.4190 (5)	0.1433 (3)	0.07374 (18)	0.0628 (11)	
O1	0.4349 (4)	0.17543 (19)	0.22135 (16)	0.0290 (8)	
O2	0.8536 (4)	0.34304 (19)	0.83524 (15)	0.0302 (8)	
O3	0.2879 (6)	0.2686 (3)	0.4573 (3)	0.0656 (13)	
O4	0.6200 (6)	0.0321 (3)	0.2472 (2)	0.0611 (13)	
O5	0.7267 (5)	0.3241 (2)	0.94652 (16)	0.0368 (9)	
N1	0.2907 (5)	0.2389 (2)	0.30914 (17)	0.0245 (9)	
N2	0.5580 (5)	0.1425 (2)	0.33825 (17)	0.0227 (9)	
N3	0.7479 (5)	0.2020 (2)	0.25942 (17)	0.0245 (9)	
N4	0.5598 (5)	0.3056 (2)	0.31532 (18)	0.0238 (9)	
N5	0.4694 (5)	0.3443 (3)	0.20092 (19)	0.0280 (10)	
N6	0.3957 (5)	0.3020 (3)	0.1265 (2)	0.0340 (11)	
N7	1.0247 (5)	0.2183 (3)	0.80379 (19)	0.0290 (10)	
N8	0.8005 (5)	0.1961 (3)	0.87833 (19)	0.0305 (10)	
N9	0.5535 (5)	0.2930 (3)	0.81949 (19)	0.0282 (9)	
N10	0.7312 (5)	0.2005 (3)	0.75325 (18)	0.0292 (10)	
N11	0.7617 (5)	0.3399 (3)	0.70462 (19)	0.0298 (10)	
N12	0.8098 (5)	0.4436 (3)	0.72442 (18)	0.0321 (10)	
C1	0.1572 (6)	0.2924 (3)	0.2925 (3)	0.0264 (11)	
C2	0.0222 (6)	0.2880 (3)	0.3239 (2)	0.0276 (11)	
C3	0.0199 (6)	0.2232 (3)	0.3735 (3)	0.0285 (11)	
C4	0.1546 (6)	0.1664 (3)	0.3905 (2)	0.0242 (10)	
C5	0.2892 (6)	0.1752 (3)	0.3580 (2)	0.0224 (10)	
C6	0.4396 (6)	0.1189 (3)	0.3733 (2)	0.0225 (10)	
C7	0.4665 (6)	0.0477 (3)	0.4167 (2)	0.0280 (11)	
C8	0.6163 (6)	0.0016 (3)	0.4248 (3)	0.0303 (12)	
C9	0.7372 (6)	0.0268 (3)	0.3894 (2)	0.0261 (11)	
C10	0.7045 (5)	0.0977 (3)	0.3450 (2)	0.0213 (10)	
C11	0.8148 (6)	0.1326 (3)	0.3011 (2)	0.0226 (10)	
C12	0.9727 (6)	0.0988 (3)	0.3004 (3)	0.0254 (11)	
C13	1.0652 (6)	0.1355 (3)	0.2560 (3)	0.0291 (11)	
C14	0.9997 (6)	0.2052 (3)	0.2143 (3)	0.0306 (12)	
C15	0.8413 (6)	0.2370 (3)	0.2175 (3)	0.0305 (12)	
C16	0.5942 (6)	0.2821 (3)	0.3756 (3)	0.0291 (11)	
C17	0.6326 (6)	0.3286 (3)	0.4037 (3)	0.0361 (13)	
C18	0.6371 (7)	0.4022 (3)	0.3692 (3)	0.0426 (14)	
C19	0.5968 (7)	0.4268 (3)	0.3081 (3)	0.0391 (14)	
C20	0.5547 (6)	0.3789 (3)	0.2815 (3)	0.0290 (12)	
C21	0.5011 (6)	0.4000 (3)	0.2174 (3)	0.0309 (12)	
C22	0.4815 (6)	0.4750 (3)	0.1764 (3)	0.0359 (13)	
C23	0.4309 (6)	0.4919 (3)	0.1185 (3)	0.0399 (14)	
C24	0.3992 (6)	0.4354 (3)	0.0988 (3)	0.0362 (14)	
C25	0.3473 (7)	0.4459 (4)	0.0382 (3)	0.0434 (15)	
C26	0.3232 (7)	0.3879 (4)	0.0234 (3)	0.0433 (15)	
C27	0.3471 (7)	0.3153 (4)	0.0696 (3)	0.0407 (14)	
C28	0.4208 (6)	0.3607 (3)	0.1415 (3)	0.0304 (12)	
C29	1.1345 (6)	0.2328 (3)	0.7631 (3)	0.0305 (12)	
C30	1.2909 (7)	0.1935 (3)	0.7740 (3)	0.0406 (14)	
C31	1.3363 (7)	0.1371 (3)	0.8297 (3)	0.0363 (13)	
C32	1.2261 (7)	0.1217 (3)	0.8730 (3)	0.0339 (12)	
C33	1.0706 (6)	0.1628 (3)	0.8593 (3)	0.0300 (11)	
C34	0.9447 (6)	0.1512 (3)	0.9025 (3)	0.0333 (12)	
C35	0.9594 (7)	0.1005 (3)	0.9615 (3)	0.0377 (13)	
C36	0.8251 (7)	0.0967 (4)	0.9947 (3)	0.0435 (14)	
C37	0.6793 (7)	0.1425 (3)	0.9694 (3)	0.0375 (13)	
C38	0.6689 (6)	0.1937 (3)	0.9108 (3)	0.0327 (12)	
C39	0.5291 (6)	0.2494 (3)	0.8768 (3)	0.0311 (12)	
C40	0.3812 (6)	0.2597 (3)	0.9014 (3)	0.0332 (12)	
C41	0.2575 (7)	0.3149 (4)	0.8659 (3)	0.0398 (13)	
C42	0.2809 (6)	0.3580 (3)	0.8079 (3)	0.0355 (13)	
C43	0.4293 (6)	0.3459 (3)	0.7857 (3)	0.0318 (12)	
C44	0.7208 (7)	0.1278 (3)	0.7813 (3)	0.0373 (13)	
C45	0.6735 (7)	0.0847 (4)	0.7506 (3)	0.0421 (14)	
C46	0.6387 (8)	0.1168 (4)	0.6890 (3)	0.0474 (16)	
C47	0.6545 (7)	0.1904 (4)	0.6593 (3)	0.0424 (14)	
C48	0.7037 (7)	0.2309 (3)	0.6919 (3)	0.0346 (12)	
C49	0.7267 (6)	0.3088 (3)	0.6652 (3)	0.0329 (12)	
C50	0.7152 (7)	0.3471 (4)	0.6015 (3)	0.0374 (13)	
C51	0.7333 (7)	0.4195 (4)	0.5790 (3)	0.0394 (14)	
C52	0.7625 (6)	0.4560 (3)	0.6195 (3)	0.0327 (12)	
C53	0.7784 (7)	0.5314 (4)	0.6016 (3)	0.0432 (15)	
C54	0.8090 (7)	0.5610 (3)	0.6442 (3)	0.0420 (14)	
C55	0.8265 (7)	0.5141 (3)	0.7052 (3)	0.0381 (13)	
C56	0.7796 (6)	0.4136 (3)	0.6823 (3)	0.0292 (11)	
C57	0.2027 (8)	0.2630 (4)	0.4995 (3)	0.0470 (15)	
C58	0.0379 (7)	0.3108 (4)	0.4911 (3)	0.0449 (15)	
C59	0.2576 (10)	0.2092 (4)	0.5619 (4)	0.063 (2)	
C60	0.7462 (9)	0.0140 (5)	0.2289 (3)	0.0539 (17)	
C61	0.8585 (9)	−0.0580 (4)	0.2678 (4)	0.066 (2)	
C62	0.7970 (9)	0.0590 (5)	0.1699 (4)	0.064 (2)	
C63	0.8122 (7)	0.3141 (3)	0.9911 (3)	0.0360 (13)	
C64	0.7708 (8)	0.2675 (4)	1.0537 (3)	0.0534 (17)	
C65	0.9533 (8)	0.3461 (4)	0.9879 (3)	0.0492 (16)	
H1	0.1561	0.3355	0.2573	0.0317*	
H2	−0.0682	0.3285	0.3120	0.0331*	
H3	−0.0732	0.2182	0.3954	0.0342*	
H4	0.1550	0.1217	0.4242	0.0290*	
H1A	0.429 (6)	0.203 (2)	0.1863 (13)	0.0348*	
H1B	0.494 (5)	0.1364 (18)	0.224 (2)	0.0348*	
H7	0.3831	0.0305	0.4409	0.0335*	
H8	0.6361	−0.0475	0.4547	0.0364*	
H9	0.8411	−0.0041	0.3954	0.0313*	
H2A	0.838 (6)	0.3835 (18)	0.8085 (15)	0.0363*	
H2B	0.809 (6)	0.348 (3)	0.8643 (15)	0.0363*	
H12	1.0171	0.0511	0.3300	0.0305*	
H13	1.1731	0.1127	0.2543	0.0349*	
H14	1.0619	0.2312	0.1838	0.0367*	
H15	0.7970	0.2855	0.1890	0.0366*	
H16	0.5917	0.2316	0.3996	0.0349*	
H17	0.6559	0.3104	0.4464	0.0433*	
H18	0.6674	0.4349	0.3873	0.0511*	
H19	0.5977	0.4774	0.2837	0.0469*	
H22	0.5042	0.5131	0.1898	0.0431*	
H23	0.4166	0.5423	0.0909	0.0479*	
H25	0.3298	0.4948	0.0080	0.0521*	
H26	0.2908	0.3949	−0.0171	0.0519*	
H27	0.3273	0.2744	0.0592	0.0489*	
H29	1.1037	0.2719	0.7250	0.0366*	
H30	1.3657	0.2050	0.7439	0.0487*	
H31	1.4430	0.1090	0.8381	0.0435*	
H32	1.2564	0.0837	0.9116	0.0406*	
H35	1.0596	0.0690	0.9788	0.0453*	
H36	0.8334	0.0623	1.0353	0.0523*	
H37	0.5871	0.1388	0.9920	0.0450*	
H40	0.3659	0.2294	0.9416	0.0399*	
H41	0.1561	0.3229	0.8819	0.0478*	
H42	0.1960	0.3957	0.7833	0.0425*	
H43	0.4449	0.3756	0.7453	0.0382*	
H44	0.7468	0.1053	0.8237	0.0448*	
H45	0.6656	0.0340	0.7718	0.0505*	
H46	0.6041	0.0890	0.6671	0.0569*	
H47	0.6319	0.2131	0.6167	0.0508*	
H50	0.6950	0.3222	0.5748	0.0449*	
H51	0.7264	0.4456	0.5364	0.0473*	
H53	0.7679	0.5618	0.5598	0.0519*	
H54	0.8183	0.6121	0.6327	0.0503*	
H55	0.8518	0.5343	0.7346	0.0457*	
H58A	0.0117	0.3399	0.4479	0.0539*	
H58B	0.0334	0.3453	0.5136	0.0539*	
H58C	−0.0390	0.2784	0.5063	0.0539*	
H59A	0.3657	0.1809	0.5615	0.0760*	
H59B	0.1854	0.1741	0.5756	0.0760*	
H59C	0.2578	0.2372	0.5896	0.0760*	
H61A	0.8022	−0.0860	0.3017	0.0791*	
H61B	0.9503	−0.0455	0.2837	0.0791*	
H61C	0.8955	−0.0891	0.2432	0.0791*	
H62A	0.7818	0.1117	0.1671	0.0771*	
H62B	0.7331	0.0558	0.1370	0.0771*	
H62C	0.9099	0.0395	0.1666	0.0771*	
H64A	0.6838	0.2431	1.0496	0.0641*	
H64B	0.7373	0.3003	1.0772	0.0641*	
H64C	0.8646	0.2289	1.0746	0.0641*	
H65A	0.9591	0.3804	0.9464	0.0591*	
H65B	1.0487	0.3055	0.9991	0.0591*	
H65C	0.9471	0.3739	1.0159	0.0591*	

### Atomic displacement parameters (Å^2^)

**Table:**

	*U*^11^	*U*^22^	*U*^33^	*U*^12^	*U*^13^	*U*^23^
Ru1	0.01521 (19)	0.0227 (2)	0.0217 (2)	0.00039 (15)	0.00309 (14)	0.00122 (15)
Ru2	0.0229 (3)	0.0280 (3)	0.0257 (3)	−0.00192 (16)	0.00797 (16)	−0.00272 (17)
P1	0.0256 (7)	0.0253 (7)	0.0252 (7)	−0.0004 (6)	0.0021 (6)	−0.0028 (6)
P2	0.0305 (8)	0.0345 (8)	0.0415 (9)	−0.0071 (6)	0.0088 (6)	−0.0098 (7)
P3	0.0263 (7)	0.0385 (8)	0.0279 (8)	0.0008 (6)	0.0037 (6)	0.0041 (6)
P4	0.0395 (8)	0.0371 (8)	0.0336 (8)	−0.0001 (7)	0.0050 (7)	−0.0026 (7)
F1	0.0315 (16)	0.0244 (15)	0.0416 (18)	−0.0014 (13)	0.0072 (13)	−0.0067 (13)
F2	0.0459 (19)	0.0286 (16)	0.0370 (18)	−0.0049 (14)	0.0021 (14)	−0.0041 (13)
F3	0.0224 (15)	0.0380 (17)	0.060 (3)	−0.0042 (13)	0.0050 (14)	−0.0167 (16)
F4	0.0329 (17)	0.0421 (18)	0.0428 (19)	−0.0097 (14)	0.0149 (14)	−0.0116 (15)
F5	0.0305 (16)	0.0410 (17)	0.0285 (16)	0.0029 (14)	0.0002 (13)	−0.0069 (14)
F6	0.060 (3)	0.0421 (18)	0.0307 (18)	0.0023 (16)	−0.0088 (15)	−0.0095 (15)
F7	0.084 (4)	0.078 (3)	0.071 (3)	0.036 (3)	0.034 (3)	0.030 (3)
F8	0.049 (3)	0.054 (3)	0.058 (3)	0.0057 (18)	0.0039 (18)	−0.0062 (18)
F9	0.044 (3)	0.103 (4)	0.124 (4)	−0.025 (3)	0.002 (3)	−0.075 (4)
F10	0.042 (3)	0.060 (3)	0.181 (6)	−0.020 (2)	0.030 (3)	−0.068 (3)
F11	0.065 (3)	0.110 (4)	0.035 (2)	0.023 (3)	0.0069 (19)	−0.017 (3)
F12	0.048 (3)	0.086 (3)	0.058 (3)	0.005 (2)	0.0107 (18)	−0.037 (3)
F13	0.0385 (19)	0.066 (3)	0.0309 (18)	0.0034 (17)	0.0089 (15)	0.0097 (16)
F14	0.0429 (19)	0.0432 (18)	0.0292 (17)	−0.0011 (15)	0.0096 (14)	−0.0016 (14)
F15	0.0434 (19)	0.0382 (17)	0.0368 (18)	−0.0006 (15)	0.0065 (15)	−0.0037 (14)
F16	0.0350 (18)	0.052 (2)	0.053 (3)	0.0171 (16)	0.0115 (16)	0.0088 (17)
F17	0.046 (2)	0.099 (3)	0.043 (3)	−0.028 (3)	0.0068 (17)	−0.020 (2)
F18	0.0345 (18)	0.0395 (18)	0.061 (3)	−0.0028 (15)	0.0072 (16)	−0.0122 (17)
F19	0.059 (3)	0.089 (3)	0.037 (2)	−0.019 (3)	0.0155 (18)	−0.018 (2)
F20	0.080 (3)	0.099 (4)	0.059 (3)	−0.031 (3)	0.030 (3)	−0.043 (3)
F21	0.081 (3)	0.055 (3)	0.078 (3)	0.031 (3)	−0.022 (3)	−0.008 (3)
F22	0.056 (3)	0.063 (3)	0.100 (4)	0.018 (2)	−0.012 (3)	−0.015 (3)
F23	0.082 (3)	0.060 (3)	0.048 (3)	−0.031 (3)	0.015 (2)	−0.0129 (19)
F24	0.058 (3)	0.067 (3)	0.066 (3)	−0.022 (2)	0.020 (2)	−0.022 (2)
O1	0.0288 (19)	0.0254 (18)	0.0280 (19)	−0.0041 (15)	−0.0003 (15)	−0.0042 (15)
O2	0.0311 (19)	0.0307 (19)	0.0241 (19)	−0.0042 (16)	0.0037 (16)	−0.0048 (15)
O3	0.053 (3)	0.066 (3)	0.073 (4)	−0.012 (3)	0.019 (3)	−0.018 (3)
O4	0.065 (4)	0.062 (3)	0.053 (3)	−0.001 (3)	−0.003 (3)	−0.023 (3)
O5	0.040 (3)	0.037 (2)	0.027 (2)	−0.0018 (17)	0.0030 (17)	−0.0078 (17)
N1	0.020 (2)	0.025 (2)	0.022 (2)	−0.0010 (17)	0.0035 (16)	−0.0017 (17)
N2	0.0160 (19)	0.027 (2)	0.021 (2)	−0.0011 (16)	−0.0006 (16)	−0.0050 (17)
N3	0.020 (2)	0.026 (2)	0.024 (2)	−0.0025 (17)	0.0023 (16)	−0.0056 (17)
N4	0.0138 (18)	0.0198 (19)	0.030 (3)	0.0022 (16)	0.0019 (16)	−0.0017 (17)
N5	0.0132 (19)	0.027 (2)	0.034 (3)	−0.0008 (17)	0.0072 (17)	−0.0004 (18)
N6	0.023 (3)	0.033 (3)	0.034 (3)	0.0010 (19)	0.0048 (19)	−0.000 (2)
N7	0.026 (3)	0.026 (2)	0.030 (3)	−0.0051 (18)	0.0105 (18)	−0.0041 (18)
N8	0.027 (3)	0.032 (3)	0.030 (3)	−0.0084 (19)	0.0063 (18)	−0.0073 (19)
N9	0.023 (2)	0.031 (3)	0.032 (3)	−0.0059 (18)	0.0065 (18)	−0.0125 (19)
N10	0.027 (3)	0.031 (3)	0.026 (3)	−0.0016 (18)	0.0076 (18)	−0.0079 (18)
N11	0.021 (2)	0.035 (3)	0.029 (3)	−0.0042 (18)	0.0059 (18)	−0.0063 (19)
N12	0.031 (3)	0.032 (3)	0.026 (3)	−0.0017 (19)	0.0019 (18)	−0.0038 (19)
C1	0.017 (3)	0.026 (3)	0.025 (3)	0.000 (2)	0.0006 (19)	0.001 (2)
C2	0.023 (3)	0.034 (3)	0.019 (3)	0.006 (2)	−0.001 (2)	−0.008 (2)
C3	0.020 (3)	0.033 (3)	0.030 (3)	−0.003 (2)	0.006 (2)	−0.010 (3)
C4	0.019 (3)	0.028 (3)	0.024 (3)	−0.007 (2)	0.0040 (19)	−0.005 (2)
C5	0.018 (3)	0.024 (3)	0.020 (3)	−0.0015 (19)	−0.0007 (18)	−0.0031 (19)
C6	0.020 (3)	0.023 (3)	0.022 (3)	−0.0030 (19)	0.0011 (19)	−0.0059 (19)
C7	0.030 (3)	0.025 (3)	0.022 (3)	−0.006 (2)	0.007 (2)	−0.001 (2)
C8	0.031 (3)	0.022 (3)	0.027 (3)	0.000 (2)	0.003 (3)	0.000 (2)
C9	0.024 (3)	0.020 (3)	0.028 (3)	0.0028 (19)	0.001 (2)	−0.004 (2)
C10	0.017 (3)	0.026 (3)	0.021 (3)	−0.0044 (19)	0.0044 (18)	−0.0087 (19)
C11	0.019 (3)	0.024 (3)	0.023 (3)	−0.0037 (19)	0.0023 (19)	−0.0055 (19)
C12	0.018 (3)	0.022 (3)	0.034 (3)	−0.0005 (19)	0.001 (2)	−0.010 (2)
C13	0.017 (3)	0.038 (3)	0.034 (3)	−0.001 (2)	0.004 (2)	−0.017 (3)
C14	0.022 (3)	0.037 (3)	0.030 (3)	−0.011 (3)	0.008 (2)	−0.006 (3)
C15	0.024 (3)	0.035 (3)	0.027 (3)	−0.003 (3)	0.006 (2)	−0.005 (3)
C16	0.023 (3)	0.021 (3)	0.035 (3)	0.001 (2)	−0.001 (2)	−0.003 (3)
C17	0.031 (3)	0.030 (3)	0.041 (4)	0.001 (3)	−0.004 (3)	−0.009 (3)
C18	0.035 (3)	0.034 (3)	0.061 (4)	−0.002 (3)	−0.003 (3)	−0.023 (3)
C19	0.030 (3)	0.024 (3)	0.053 (4)	−0.001 (3)	0.005 (3)	−0.004 (3)
C20	0.019 (3)	0.018 (3)	0.043 (3)	−0.0002 (19)	0.009 (3)	−0.004 (3)
C21	0.017 (3)	0.031 (3)	0.033 (3)	0.002 (2)	0.005 (2)	−0.000 (3)
C22	0.027 (3)	0.024 (3)	0.047 (4)	−0.003 (3)	0.006 (3)	−0.001 (3)
C23	0.027 (3)	0.030 (3)	0.040 (4)	0.004 (3)	0.009 (3)	0.011 (3)
C24	0.016 (3)	0.035 (3)	0.038 (3)	0.002 (3)	0.008 (3)	0.008 (3)
C25	0.029 (3)	0.046 (4)	0.032 (3)	0.002 (3)	0.003 (3)	0.010 (3)
C26	0.032 (3)	0.054 (4)	0.029 (3)	−0.004 (3)	−0.003 (3)	0.000 (3)
C27	0.035 (3)	0.045 (4)	0.029 (3)	−0.002 (3)	0.001 (3)	−0.001 (3)
C28	0.015 (3)	0.037 (3)	0.024 (3)	0.001 (2)	0.0036 (19)	0.005 (3)
C29	0.024 (3)	0.027 (3)	0.039 (3)	−0.007 (2)	0.012 (3)	−0.008 (3)
C30	0.033 (3)	0.031 (3)	0.058 (4)	−0.009 (3)	0.025 (3)	−0.015 (3)
C31	0.028 (3)	0.031 (3)	0.045 (4)	0.000 (3)	0.007 (3)	−0.009 (3)
C32	0.036 (3)	0.025 (3)	0.036 (3)	−0.003 (3)	0.004 (3)	−0.006 (3)
C33	0.029 (3)	0.025 (3)	0.030 (3)	−0.005 (2)	0.006 (3)	−0.003 (2)
C34	0.029 (3)	0.029 (3)	0.035 (3)	−0.002 (3)	0.004 (3)	−0.005 (3)
C35	0.041 (3)	0.033 (3)	0.028 (3)	−0.003 (3)	0.006 (3)	0.000 (3)
C36	0.046 (4)	0.044 (4)	0.028 (3)	−0.004 (3)	0.009 (3)	−0.000 (3)
C37	0.039 (3)	0.039 (3)	0.033 (3)	−0.012 (3)	0.015 (3)	−0.008 (3)
C38	0.025 (3)	0.042 (3)	0.034 (3)	−0.009 (3)	0.011 (3)	−0.015 (3)
C39	0.032 (3)	0.034 (3)	0.028 (3)	−0.010 (3)	0.010 (3)	−0.010 (3)
C40	0.029 (3)	0.044 (3)	0.033 (3)	−0.011 (3)	0.011 (3)	−0.019 (3)
C41	0.029 (3)	0.051 (4)	0.045 (4)	−0.009 (3)	0.005 (3)	−0.023 (3)
C42	0.028 (3)	0.040 (3)	0.043 (4)	−0.003 (3)	−0.002 (3)	−0.023 (3)
C43	0.026 (3)	0.032 (3)	0.034 (3)	0.000 (3)	0.001 (3)	−0.010 (3)
C44	0.037 (3)	0.035 (3)	0.036 (3)	−0.000 (3)	0.015 (3)	−0.012 (3)
C45	0.050 (4)	0.037 (3)	0.042 (4)	−0.004 (3)	0.021 (3)	−0.020 (3)
C46	0.060 (4)	0.047 (4)	0.045 (4)	−0.016 (3)	0.024 (3)	−0.026 (3)
C47	0.041 (4)	0.055 (4)	0.028 (3)	−0.006 (3)	0.013 (3)	−0.014 (3)
C48	0.032 (3)	0.041 (3)	0.031 (3)	−0.005 (3)	0.011 (3)	−0.014 (3)
C49	0.025 (3)	0.040 (3)	0.026 (3)	−0.004 (3)	0.011 (3)	−0.004 (3)
C50	0.034 (3)	0.047 (4)	0.026 (3)	−0.008 (3)	0.009 (3)	−0.006 (3)
C51	0.030 (3)	0.053 (4)	0.022 (3)	−0.006 (3)	0.005 (3)	0.001 (3)
C52	0.022 (3)	0.037 (3)	0.026 (3)	−0.001 (3)	0.003 (2)	0.002 (3)
C53	0.038 (4)	0.044 (4)	0.031 (3)	−0.006 (3)	0.005 (3)	0.006 (3)
C54	0.051 (4)	0.032 (3)	0.037 (4)	−0.010 (3)	0.003 (3)	−0.002 (3)
C55	0.041 (4)	0.035 (3)	0.032 (3)	−0.008 (3)	0.005 (3)	−0.005 (3)
C56	0.022 (3)	0.034 (3)	0.023 (3)	−0.002 (2)	0.005 (2)	−0.001 (3)
C57	0.057 (4)	0.044 (4)	0.045 (4)	−0.023 (3)	0.001 (3)	−0.015 (3)
C58	0.039 (4)	0.048 (4)	0.051 (4)	−0.012 (3)	0.002 (3)	−0.019 (3)
C59	0.080 (6)	0.041 (4)	0.063 (5)	−0.007 (4)	−0.019 (4)	−0.013 (4)
C60	0.050 (4)	0.077 (5)	0.045 (4)	−0.018 (4)	0.000 (4)	−0.032 (4)
C61	0.067 (5)	0.060 (5)	0.077 (6)	0.003 (4)	−0.011 (4)	−0.041 (4)
C62	0.062 (5)	0.089 (6)	0.054 (5)	−0.023 (5)	0.013 (4)	−0.036 (4)
C63	0.033 (3)	0.029 (3)	0.039 (4)	0.001 (3)	0.004 (3)	−0.008 (3)
C64	0.051 (4)	0.067 (5)	0.035 (4)	−0.011 (4)	0.011 (3)	−0.009 (3)
C65	0.049 (4)	0.047 (4)	0.047 (4)	−0.011 (3)	0.006 (3)	−0.010 (3)

### Geometric parameters (Å, °)

**Table:**

Ru1—O1	2.150 (5)	C32—C33	1.387 (7)
Ru1—N1	2.073 (4)	C33—C34	1.473 (8)
Ru1—N2	1.956 (4)	C34—C35	1.386 (7)
Ru1—N3	2.076 (4)	C35—C36	1.387 (9)
Ru1—N4	2.019 (5)	C36—C37	1.384 (8)
Ru1—N5	2.098 (4)	C37—C38	1.384 (7)
Ru2—O2	2.138 (5)	C38—C39	1.466 (7)
Ru2—N7	2.066 (4)	C39—C40	1.397 (8)
Ru2—N8	1.952 (4)	C40—C41	1.383 (7)
Ru2—N9	2.072 (4)	C41—C42	1.370 (8)
Ru2—N10	2.023 (6)	C42—C43	1.380 (8)
Ru2—N11	2.097 (4)	C44—C45	1.393 (11)
P1—F1	1.597 (4)	C45—C46	1.373 (9)
P1—F2	1.605 (4)	C46—C47	1.387 (9)
P1—F3	1.601 (4)	C47—C48	1.391 (10)
P1—F4	1.599 (4)	C48—C49	1.472 (8)
P1—F5	1.602 (4)	C49—C50	1.419 (7)
P1—F6	1.586 (4)	C50—C51	1.358 (9)
P2—F7	1.559 (5)	C51—C52	1.416 (10)
P2—F8	1.601 (4)	C52—C53	1.407 (9)
P2—F9	1.576 (6)	C52—C56	1.413 (7)
P2—F10	1.596 (6)	C53—C54	1.364 (10)
P2—F11	1.576 (5)	C54—C55	1.400 (8)
P2—F12	1.592 (5)	C57—C58	1.501 (9)
P3—F13	1.600 (4)	C57—C59	1.493 (9)
P3—F14	1.586 (4)	C60—C61	1.521 (9)
P3—F15	1.590 (4)	C60—C62	1.477 (10)
P3—F16	1.593 (4)	C63—C64	1.521 (8)
P3—F17	1.598 (5)	C63—C65	1.455 (10)
P3—F18	1.610 (5)	O1—H1A	0.81 (3)
P4—F19	1.590 (5)	O1—H1B	0.81 (4)
P4—F20	1.577 (6)	O2—H2A	0.80 (3)
P4—F21	1.576 (4)	O2—H2B	0.81 (4)
P4—F22	1.581 (4)	C1—H1	0.950
P4—F23	1.603 (5)	C2—H2	0.950
P4—F24	1.599 (5)	C3—H3	0.950
O3—C57	1.215 (9)	C4—H4	0.950
O4—C60	1.194 (9)	C7—H7	0.950
O5—C63	1.222 (8)	C8—H8	0.950
N1—C1	1.339 (6)	C9—H9	0.950
N1—C5	1.368 (6)	C12—H12	0.950
N2—C6	1.353 (6)	C13—H13	0.950
N2—C10	1.353 (6)	C14—H14	0.950
N3—C11	1.375 (5)	C15—H15	0.950
N3—C15	1.347 (7)	C16—H16	0.950
N4—C16	1.348 (7)	C17—H17	0.950
N4—C20	1.359 (6)	C18—H18	0.950
N5—C21	1.339 (8)	C19—H19	0.950
N5—C28	1.372 (7)	C22—H22	0.950
N6—C27	1.327 (8)	C23—H23	0.950
N6—C28	1.354 (9)	C25—H25	0.950
N7—C29	1.337 (7)	C26—H26	0.950
N7—C33	1.374 (6)	C27—H27	0.950
N8—C34	1.361 (6)	C29—H29	0.950
N8—C38	1.357 (7)	C30—H30	0.950
N9—C39	1.362 (7)	C31—H31	0.950
N9—C43	1.355 (6)	C32—H32	0.950
N10—C44	1.352 (7)	C35—H35	0.950
N10—C48	1.360 (7)	C36—H36	0.950
N11—C49	1.332 (9)	C37—H37	0.950
N11—C56	1.380 (7)	C40—H40	0.950
N12—C55	1.317 (8)	C41—H41	0.950
N12—C56	1.360 (8)	C42—H42	0.950
C1—C2	1.368 (7)	C43—H43	0.950
C2—C3	1.391 (6)	C44—H44	0.950
C3—C4	1.380 (6)	C45—H45	0.950
C4—C5	1.385 (7)	C46—H46	0.950
C5—C6	1.470 (6)	C47—H47	0.950
C6—C7	1.379 (6)	C50—H50	0.950
C7—C8	1.383 (7)	C51—H51	0.950
C8—C9	1.383 (7)	C53—H53	0.950
C9—C10	1.384 (6)	C54—H54	0.950
C10—C11	1.474 (7)	C55—H55	0.950
C11—C12	1.385 (6)	C58—H58A	0.980
C12—C13	1.388 (7)	C58—H58B	0.980
C13—C14	1.376 (6)	C58—H58C	0.980
C14—C15	1.389 (7)	C59—H59A	0.980
C16—C17	1.373 (9)	C59—H59B	0.980
C17—C18	1.383 (8)	C59—H59C	0.980
C18—C19	1.373 (9)	C61—H61A	0.980
C19—C20	1.385 (10)	C61—H61B	0.980
C20—C21	1.472 (8)	C61—H61C	0.980
C21—C22	1.415 (7)	C62—H62A	0.980
C22—C23	1.340 (9)	C62—H62B	0.980
C23—C24	1.399 (10)	C62—H62C	0.980
C24—C25	1.433 (9)	C64—H64A	0.980
C24—C28	1.423 (7)	C64—H64B	0.980
C25—C26	1.337 (11)	C64—H64C	0.980
C26—C27	1.427 (8)	C65—H65A	0.980
C29—C30	1.382 (7)	C65—H65B	0.980
C30—C31	1.383 (7)	C65—H65C	0.980
C31—C32	1.381 (8)		
			
O1—Ru1—N1	86.71 (17)	N7—C33—C34	115.4 (4)
O1—Ru1—N2	84.64 (16)	C32—C33—C34	123.2 (4)
O1—Ru1—N3	91.56 (16)	N8—C34—C33	112.8 (4)
O1—Ru1—N4	174.62 (13)	N8—C34—C35	119.9 (5)
O1—Ru1—N5	98.06 (16)	C33—C34—C35	127.3 (5)
N1—Ru1—N2	79.53 (14)	C34—C35—C36	118.7 (5)
N1—Ru1—N3	158.89 (13)	C35—C36—C37	120.6 (5)
N1—Ru1—N4	89.74 (17)	C36—C37—C38	119.4 (5)
N1—Ru1—N5	102.52 (14)	N8—C38—C37	119.5 (5)
N2—Ru1—N3	79.36 (14)	N8—C38—C39	112.4 (4)
N2—Ru1—N4	98.70 (17)	C37—C38—C39	128.1 (5)
N2—Ru1—N5	176.67 (18)	N9—C39—C38	115.5 (5)
N3—Ru1—N4	93.20 (17)	N9—C39—C40	121.4 (4)
N3—Ru1—N5	98.56 (14)	C38—C39—C40	123.0 (5)
N4—Ru1—N5	78.76 (17)	C39—C40—C41	118.6 (5)
O2—Ru2—N7	86.74 (17)	C40—C41—C42	120.2 (5)
O2—Ru2—N8	86.26 (18)	C41—C42—C43	119.1 (5)
O2—Ru2—N9	90.55 (18)	N9—C43—C42	122.2 (5)
O2—Ru2—N10	175.87 (15)	N10—C44—C45	122.8 (5)
O2—Ru2—N11	98.32 (17)	C44—C45—C46	118.6 (6)
N7—Ru2—N8	80.22 (16)	C45—C46—C47	119.3 (7)
N7—Ru2—N9	159.48 (15)	C46—C47—C48	119.9 (6)
N7—Ru2—N10	91.26 (18)	N10—C48—C47	121.1 (5)
N7—Ru2—N11	100.61 (16)	N10—C48—C49	114.2 (6)
N8—Ru2—N9	79.30 (16)	C47—C48—C49	124.7 (5)
N8—Ru2—N10	96.97 (19)	N11—C49—C48	115.6 (5)
N8—Ru2—N11	175.4 (2)	N11—C49—C50	122.7 (6)
N9—Ru2—N10	92.58 (19)	C48—C49—C50	121.7 (6)
N9—Ru2—N11	99.91 (15)	C49—C50—C51	119.6 (7)
N10—Ru2—N11	78.48 (18)	C50—C51—C52	119.5 (5)
F1—P1—F2	179.21 (19)	C51—C52—C53	124.6 (5)
F1—P1—F3	90.49 (18)	C51—C52—C56	118.1 (5)
F1—P1—F4	90.17 (17)	C53—C52—C56	117.3 (6)
F1—P1—F5	89.60 (17)	C52—C53—C54	120.1 (5)
F1—P1—F6	90.81 (19)	C53—C54—C55	118.2 (6)
F2—P1—F3	89.23 (18)	N12—C55—C54	124.0 (7)
F2—P1—F4	90.10 (18)	N11—C56—N12	115.9 (4)
F2—P1—F5	89.66 (18)	N11—C56—C52	121.9 (6)
F2—P1—F6	89.94 (18)	N12—C56—C52	122.2 (5)
F3—P1—F4	178.6 (3)	O3—C57—C58	121.3 (6)
F3—P1—F5	90.04 (18)	O3—C57—C59	121.7 (6)
F3—P1—F6	91.23 (19)	C58—C57—C59	117.1 (6)
F4—P1—F5	88.72 (17)	O4—C60—C61	118.7 (6)
F4—P1—F6	90.01 (19)	O4—C60—C62	122.5 (6)
F5—P1—F6	178.7 (2)	C61—C60—C62	118.9 (7)
F7—P2—F8	177.8 (3)	O5—C63—C64	119.8 (6)
F7—P2—F9	93.0 (3)	O5—C63—C65	123.1 (5)
F7—P2—F10	92.2 (3)	C64—C63—C65	117.1 (6)
F7—P2—F11	92.8 (3)	Ru1—O1—H1A	104 (4)
F7—P2—F12	88.9 (3)	Ru1—O1—H1B	117 (4)
F8—P2—F9	88.9 (3)	H1A—O1—H1B	107 (5)
F8—P2—F10	85.8 (3)	Ru2—O2—H2A	105 (3)
F8—P2—F11	88.2 (3)	Ru2—O2—H2B	118 (4)
F8—P2—F12	90.1 (2)	H2A—O2—H2B	108 (4)
F9—P2—F10	173.2 (3)	N1—C1—H1	118.404
F9—P2—F11	91.6 (3)	C2—C1—H1	118.406
F9—P2—F12	87.3 (3)	C1—C2—H2	120.681
F10—P2—F11	92.5 (3)	C3—C2—H2	120.665
F10—P2—F12	88.4 (3)	C2—C3—H3	120.410
F11—P2—F12	178.0 (3)	C4—C3—H3	120.407
F13—P3—F14	179.4 (3)	C3—C4—H4	120.308
F13—P3—F15	90.11 (18)	C5—C4—H4	120.312
F13—P3—F16	89.73 (19)	C6—C7—H7	120.349
F13—P3—F17	90.1 (2)	C8—C7—H7	120.340
F13—P3—F18	89.3 (2)	C7—C8—H8	119.925
F14—P3—F15	90.09 (17)	C9—C8—H8	119.939
F14—P3—F16	90.07 (18)	C8—C9—H9	120.530
F14—P3—F17	90.4 (2)	C10—C9—H9	120.536
F14—P3—F18	90.2 (2)	C11—C12—H12	120.469
F15—P3—F16	179.84 (19)	C13—C12—H12	120.465
F15—P3—F17	90.4 (3)	C12—C13—H13	120.190
F15—P3—F18	89.94 (19)	C14—C13—H13	120.187
F16—P3—F17	89.7 (3)	C13—C14—H14	120.537
F16—P3—F18	90.0 (2)	C15—C14—H14	120.552
F17—P3—F18	179.28 (19)	N3—C15—H15	118.627
F19—P4—F20	179.0 (3)	C14—C15—H15	118.633
F19—P4—F21	90.3 (3)	N4—C16—H16	118.811
F19—P4—F22	89.1 (3)	C17—C16—H16	118.803
F19—P4—F23	89.4 (3)	C16—C17—H17	120.381
F19—P4—F24	90.1 (3)	C18—C17—H17	120.384
F20—P4—F21	90.5 (3)	C17—C18—H18	120.832
F20—P4—F22	90.0 (3)	C19—C18—H18	120.837
F20—P4—F23	90.1 (3)	C18—C19—H19	119.541
F20—P4—F24	90.4 (3)	C20—C19—H19	119.554
F21—P4—F22	178.9 (3)	C21—C22—H22	120.106
F21—P4—F23	90.0 (3)	C23—C22—H22	120.121
F21—P4—F24	90.2 (3)	C22—C23—H23	120.092
F22—P4—F23	89.0 (3)	C24—C23—H23	120.069
F22—P4—F24	90.8 (3)	C24—C25—H25	119.562
F23—P4—F24	179.5 (3)	C26—C25—H25	119.545
Ru1—N1—C1	128.5 (3)	C25—C26—H26	120.724
Ru1—N1—C5	113.2 (3)	C27—C26—H26	120.706
C1—N1—C5	118.3 (4)	N6—C27—H27	118.385
Ru1—N2—C6	119.0 (3)	C26—C27—H27	118.393
Ru1—N2—C10	119.3 (3)	N7—C29—H29	118.547
C6—N2—C10	121.4 (4)	C30—C29—H29	118.550
Ru1—N3—C11	113.6 (3)	C29—C30—H30	120.688
Ru1—N3—C15	128.6 (3)	C31—C30—H30	120.694
C11—N3—C15	117.9 (4)	C30—C31—H31	120.114
Ru1—N4—C16	124.2 (4)	C32—C31—H31	120.124
Ru1—N4—C20	116.9 (4)	C31—C32—H32	120.501
C16—N4—C20	118.9 (5)	C33—C32—H32	120.509
Ru1—N5—C21	114.2 (4)	C34—C35—H35	120.647
Ru1—N5—C28	127.6 (4)	C36—C35—H35	120.657
C21—N5—C28	118.2 (4)	C35—C36—H36	119.697
C27—N6—C28	118.2 (5)	C37—C36—H36	119.700
Ru2—N7—C29	128.9 (3)	C36—C37—H37	120.291
Ru2—N7—C33	112.8 (4)	C38—C37—H37	120.303
C29—N7—C33	118.3 (4)	C39—C40—H40	120.712
Ru2—N8—C34	118.7 (4)	C41—C40—H40	120.707
Ru2—N8—C38	119.4 (3)	C40—C41—H41	119.915
C34—N8—C38	121.8 (4)	C42—C41—H41	119.916
Ru2—N9—C39	113.4 (3)	C41—C42—H42	120.416
Ru2—N9—C43	128.1 (4)	C43—C42—H42	120.442
C39—N9—C43	118.5 (5)	N9—C43—H43	118.926
Ru2—N10—C44	124.9 (4)	C42—C43—H43	118.919
Ru2—N10—C48	116.9 (4)	N10—C44—H44	118.613
C44—N10—C48	118.2 (6)	C45—C44—H44	118.599
Ru2—N11—C49	114.7 (4)	C44—C45—H45	120.684
Ru2—N11—C56	127.1 (4)	C46—C45—H45	120.695
C49—N11—C56	118.2 (5)	C45—C46—H46	120.369
C55—N12—C56	118.1 (5)	C47—C46—H46	120.375
N1—C1—C2	123.2 (4)	C46—C47—H47	120.055
C1—C2—C3	118.7 (4)	C48—C47—H47	120.076
C2—C3—C4	119.2 (5)	C49—C50—H50	120.220
C3—C4—C5	119.4 (4)	C51—C50—H50	120.221
N1—C5—C4	121.2 (4)	C50—C51—H51	120.240
N1—C5—C6	115.1 (4)	C52—C51—H51	120.235
C4—C5—C6	123.6 (4)	C52—C53—H53	119.931
N2—C6—C5	112.9 (4)	C54—C53—H53	119.941
N2—C6—C7	120.1 (4)	C53—C54—H54	120.871
C5—C6—C7	127.0 (5)	C55—C54—H54	120.886
C6—C7—C8	119.3 (5)	N12—C55—H55	117.999
C7—C8—C9	120.1 (4)	C54—C55—H55	117.999
C8—C9—C10	118.9 (4)	C57—C58—H58A	109.475
N2—C10—C9	120.2 (4)	C57—C58—H58B	109.468
N2—C10—C11	113.1 (4)	C57—C58—H58C	109.478
C9—C10—C11	126.8 (4)	H58A—C58—H58B	109.452
N3—C11—C10	114.6 (4)	H58A—C58—H58C	109.481
N3—C11—C12	121.8 (4)	H58B—C58—H58C	109.473
C10—C11—C12	123.7 (4)	C57—C59—H59A	109.461
C11—C12—C13	119.1 (4)	C57—C59—H59B	109.463
C12—C13—C14	119.6 (5)	C57—C59—H59C	109.467
C13—C14—C15	118.9 (5)	H59A—C59—H59B	109.480
N3—C15—C14	122.7 (4)	H59A—C59—H59C	109.472
N4—C16—C17	122.4 (5)	H59B—C59—H59C	109.484
C16—C17—C18	119.2 (6)	C60—C61—H61A	109.473
C17—C18—C19	118.3 (7)	C60—C61—H61B	109.469
C18—C19—C20	120.9 (5)	C60—C61—H61C	109.482
N4—C20—C19	120.1 (5)	H61A—C61—H61B	109.477
N4—C20—C21	114.2 (5)	H61A—C61—H61C	109.458
C19—C20—C21	125.7 (5)	H61B—C61—H61C	109.468
N5—C21—C20	115.8 (4)	C60—C62—H62A	109.464
N5—C21—C22	122.5 (5)	C60—C62—H62B	109.471
C20—C21—C22	121.7 (6)	C60—C62—H62C	109.469
C21—C22—C23	119.8 (6)	H62A—C62—H62B	109.484
C22—C23—C24	119.8 (5)	H62A—C62—H62C	109.466
C23—C24—C25	125.2 (5)	H62B—C62—H62C	109.473
C23—C24—C28	118.6 (6)	C63—C64—H64A	109.475
C25—C24—C28	116.2 (6)	C63—C64—H64B	109.471
C24—C25—C26	120.9 (5)	C63—C64—H64C	109.469
C25—C26—C27	118.6 (6)	H64A—C64—H64B	109.471
N6—C27—C26	123.2 (7)	H64A—C64—H64C	109.479
N5—C28—N6	116.1 (4)	H64B—C64—H64C	109.463
N5—C28—C24	121.0 (6)	C63—C65—H65A	109.473
N6—C28—C24	122.9 (5)	C63—C65—H65B	109.467
N7—C29—C30	122.9 (5)	C63—C65—H65C	109.474
C29—C30—C31	118.6 (6)	H65A—C65—H65B	109.478
C30—C31—C32	119.8 (5)	H65A—C65—H65C	109.474
C31—C32—C33	119.0 (5)	H65B—C65—H65C	109.463
N7—C33—C32	121.4 (5)		
			
O1—Ru1—N1—C1	−97.1 (4)	Ru2—N7—C33—C34	−1.9 (6)
O1—Ru1—N1—C5	84.2 (3)	C29—N7—C33—C32	−0.8 (9)
O1—Ru1—N2—C6	−84.0 (4)	C29—N7—C33—C34	178.3 (5)
O1—Ru1—N2—C10	89.7 (4)	C33—N7—C29—C30	1.3 (9)
O1—Ru1—N3—C11	−83.4 (3)	Ru2—N8—C34—C33	−2.6 (7)
O1—Ru1—N3—C15	97.2 (4)	Ru2—N8—C34—C35	178.7 (4)
O1—Ru1—N5—C21	178.3 (3)	Ru2—N8—C38—C37	180.0 (4)
O1—Ru1—N5—C28	−4.0 (3)	Ru2—N8—C38—C39	−0.6 (7)
N1—Ru1—N2—C6	3.6 (3)	C34—N8—C38—C37	−2.3 (9)
N1—Ru1—N2—C10	177.3 (4)	C34—N8—C38—C39	177.1 (5)
N2—Ru1—N1—C1	177.7 (4)	C38—N8—C34—C33	179.7 (5)
N2—Ru1—N1—C5	−1.0 (3)	C38—N8—C34—C35	1.0 (9)
N1—Ru1—N3—C11	1.5 (8)	Ru2—N9—C39—C38	−0.1 (7)
N1—Ru1—N3—C15	−177.9 (4)	Ru2—N9—C39—C40	177.9 (4)
N3—Ru1—N1—C1	177.1 (4)	Ru2—N9—C43—C42	−178.1 (4)
N3—Ru1—N1—C5	−1.7 (8)	C39—N9—C43—C42	1.6 (9)
N1—Ru1—N4—C16	71.9 (3)	C43—N9—C39—C38	−179.8 (5)
N1—Ru1—N4—C20	−106.6 (3)	C43—N9—C39—C40	−1.8 (9)
N4—Ru1—N1—C1	78.9 (4)	Ru2—N10—C44—C45	176.0 (3)
N4—Ru1—N1—C5	−99.9 (3)	Ru2—N10—C48—C47	−175.4 (3)
N1—Ru1—N5—C21	89.9 (3)	Ru2—N10—C48—C49	3.2 (6)
N1—Ru1—N5—C28	−92.4 (3)	C44—N10—C48—C47	4.7 (7)
N5—Ru1—N1—C1	0.4 (5)	C44—N10—C48—C49	−176.7 (4)
N5—Ru1—N1—C5	−178.3 (3)	C48—N10—C44—C45	−4.1 (7)
N2—Ru1—N3—C11	0.9 (3)	Ru2—N11—C49—C48	2.6 (5)
N2—Ru1—N3—C15	−178.5 (4)	Ru2—N11—C49—C50	−176.2 (3)
N3—Ru1—N2—C6	−176.6 (4)	Ru2—N11—C56—N12	−2.8 (6)
N3—Ru1—N2—C10	−2.9 (3)	Ru2—N11—C56—C52	179.1 (3)
N2—Ru1—N4—C16	−7.5 (3)	C49—N11—C56—N12	177.8 (4)
N2—Ru1—N4—C20	174.0 (3)	C49—N11—C56—C52	−0.3 (7)
N4—Ru1—N2—C6	91.7 (4)	C56—N11—C49—C48	−178.0 (4)
N4—Ru1—N2—C10	−94.5 (4)	C56—N11—C49—C50	3.3 (7)
N3—Ru1—N4—C16	−87.2 (3)	C55—N12—C56—N11	−179.4 (4)
N3—Ru1—N4—C20	94.3 (3)	C55—N12—C56—C52	−1.4 (7)
N4—Ru1—N3—C11	99.1 (3)	C56—N12—C55—C54	2.5 (8)
N4—Ru1—N3—C15	−80.3 (4)	N1—C1—C2—C3	−3.5 (9)
N3—Ru1—N5—C21	−88.9 (3)	C1—C2—C3—C4	1.6 (9)
N3—Ru1—N5—C28	88.8 (3)	C2—C3—C4—C5	0.3 (9)
N5—Ru1—N3—C11	178.2 (3)	C3—C4—C5—N1	−0.5 (9)
N5—Ru1—N3—C15	−1.2 (4)	C3—C4—C5—C6	178.5 (5)
N4—Ru1—N5—C21	2.7 (3)	N1—C5—C6—N2	4.3 (7)
N4—Ru1—N5—C28	−179.6 (4)	N1—C5—C6—C7	−174.1 (5)
N5—Ru1—N4—C16	174.7 (3)	C4—C5—C6—N2	−174.9 (5)
N5—Ru1—N4—C20	−3.8 (3)	C4—C5—C6—C7	6.7 (9)
O2—Ru2—N7—C29	−93.1 (4)	N2—C6—C7—C8	1.2 (8)
O2—Ru2—N7—C33	87.2 (3)	C5—C6—C7—C8	179.6 (5)
O2—Ru2—N8—C34	−86.0 (4)	C6—C7—C8—C9	−0.2 (9)
O2—Ru2—N8—C38	91.7 (4)	C7—C8—C9—C10	−1.7 (9)
O2—Ru2—N9—C39	−86.3 (3)	C8—C9—C10—N2	2.6 (8)
O2—Ru2—N9—C43	93.4 (4)	C8—C9—C10—C11	−176.7 (5)
O2—Ru2—N11—C49	176.6 (3)	N2—C10—C11—N3	−3.3 (7)
O2—Ru2—N11—C56	−2.8 (3)	N2—C10—C11—C12	177.8 (5)
N7—Ru2—N8—C34	1.3 (4)	C9—C10—C11—N3	176.0 (5)
N7—Ru2—N8—C38	179.1 (4)	C9—C10—C11—C12	−2.9 (9)
N8—Ru2—N7—C29	−179.9 (5)	N3—C11—C12—C13	−0.8 (9)
N8—Ru2—N7—C33	0.4 (3)	C10—C11—C12—C13	178.1 (5)
N7—Ru2—N9—C39	−4.1 (8)	C11—C12—C13—C14	1.3 (9)
N7—Ru2—N9—C43	175.6 (4)	C12—C13—C14—C15	−0.5 (9)
N9—Ru2—N7—C29	−175.9 (4)	C13—C14—C15—N3	−0.9 (10)
N9—Ru2—N7—C33	4.4 (8)	N4—C16—C17—C18	−0.2 (7)
N7—Ru2—N10—C44	77.8 (3)	C16—C17—C18—C19	2.4 (8)
N7—Ru2—N10—C48	−102.0 (3)	C17—C18—C19—C20	−0.9 (8)
N10—Ru2—N7—C29	83.3 (4)	C18—C19—C20—N4	−3.0 (8)
N10—Ru2—N7—C33	−96.4 (3)	C18—C19—C20—C21	176.5 (5)
N7—Ru2—N11—C49	88.4 (3)	N4—C20—C21—N5	−1.8 (6)
N7—Ru2—N11—C56	−91.0 (4)	N4—C20—C21—C22	176.9 (4)
N11—Ru2—N7—C29	4.8 (5)	C19—C20—C21—N5	178.7 (5)
N11—Ru2—N7—C33	−174.9 (3)	C19—C20—C21—C22	−2.5 (8)
N8—Ru2—N9—C39	−0.2 (4)	N5—C21—C22—C23	−0.6 (8)
N8—Ru2—N9—C43	179.5 (5)	C20—C21—C22—C23	−179.3 (4)
N9—Ru2—N8—C34	−177.3 (4)	C21—C22—C23—C24	−0.6 (8)
N9—Ru2—N8—C38	0.5 (4)	C22—C23—C24—C25	−178.9 (5)
N8—Ru2—N10—C44	−2.5 (4)	C22—C23—C24—C28	0.4 (8)
N8—Ru2—N10—C48	177.7 (3)	C23—C24—C25—C26	178.6 (5)
N10—Ru2—N8—C34	91.4 (4)	C23—C24—C28—N5	1.0 (7)
N10—Ru2—N8—C38	−90.9 (4)	C23—C24—C28—N6	−178.9 (4)
N9—Ru2—N10—C44	−82.0 (3)	C25—C24—C28—N5	−179.7 (4)
N9—Ru2—N10—C48	98.2 (3)	C25—C24—C28—N6	0.4 (7)
N10—Ru2—N9—C39	96.4 (4)	C28—C24—C25—C26	−0.7 (7)
N10—Ru2—N9—C43	−83.9 (4)	C24—C25—C26—C27	1.2 (8)
N9—Ru2—N11—C49	−91.3 (3)	C25—C26—C27—N6	−1.5 (8)
N9—Ru2—N11—C56	89.3 (4)	N7—C29—C30—C31	−0.6 (10)
N11—Ru2—N9—C39	175.2 (3)	C29—C30—C31—C32	−0.6 (10)
N11—Ru2—N9—C43	−5.1 (5)	C30—C31—C32—C33	1.0 (10)
N10—Ru2—N11—C49	−0.7 (3)	C31—C32—C33—N7	−0.3 (10)
N10—Ru2—N11—C56	179.9 (4)	C31—C32—C33—C34	−179.4 (6)
N11—Ru2—N10—C44	178.4 (4)	N7—C33—C34—N8	3.0 (8)
N11—Ru2—N10—C48	−1.4 (3)	N7—C33—C34—C35	−178.5 (6)
Ru1—N1—C1—C2	−175.4 (4)	C32—C33—C34—N8	−177.9 (6)
Ru1—N1—C5—C4	177.7 (4)	C32—C33—C34—C35	0.6 (10)
Ru1—N1—C5—C6	−1.5 (6)	N8—C34—C35—C36	0.1 (10)
C1—N1—C5—C4	−1.2 (8)	C33—C34—C35—C36	−178.3 (6)
C1—N1—C5—C6	179.7 (5)	C34—C35—C36—C37	0.2 (10)
C5—N1—C1—C2	3.3 (8)	C35—C36—C37—C38	−1.5 (10)
Ru1—N2—C6—C5	−5.3 (6)	C36—C37—C38—N8	2.6 (10)
Ru1—N2—C6—C7	173.2 (4)	C36—C37—C38—C39	−176.7 (6)
Ru1—N2—C10—C9	−175.1 (3)	N8—C38—C39—N9	0.4 (8)
Ru1—N2—C10—C11	4.2 (6)	N8—C38—C39—C40	−177.5 (5)
C6—N2—C10—C9	−1.5 (8)	C37—C38—C39—N9	179.8 (6)
C6—N2—C10—C11	177.8 (5)	C37—C38—C39—C40	1.8 (11)
C10—N2—C6—C5	−178.9 (5)	N9—C39—C40—C41	0.9 (10)
C10—N2—C6—C7	−0.4 (8)	C38—C39—C40—C41	178.7 (6)
Ru1—N3—C11—C10	1.0 (6)	C39—C40—C41—C42	0.2 (10)
Ru1—N3—C11—C12	−180.0 (4)	C40—C41—C42—C43	−0.4 (11)
Ru1—N3—C15—C14	−179.2 (4)	C41—C42—C43—N9	−0.5 (10)
C11—N3—C15—C14	1.4 (9)	N10—C44—C45—C46	1.1 (8)
C15—N3—C11—C10	−179.5 (5)	C44—C45—C46—C47	1.3 (9)
C15—N3—C11—C12	−0.5 (8)	C45—C46—C47—C48	−0.7 (9)
Ru1—N4—C16—C17	177.9 (3)	C46—C47—C48—N10	−2.4 (8)
Ru1—N4—C20—C19	−176.2 (3)	C46—C47—C48—C49	179.2 (5)
Ru1—N4—C20—C21	4.2 (5)	N10—C48—C49—N11	−3.8 (7)
C16—N4—C20—C19	5.1 (6)	N10—C48—C49—C50	175.0 (4)
C16—N4—C20—C21	−174.4 (4)	C47—C48—C49—N11	174.8 (5)
C20—N4—C16—C17	−3.6 (7)	C47—C48—C49—C50	−6.5 (8)
Ru1—N5—C21—C20	−1.3 (5)	N11—C49—C50—C51	−3.0 (8)
Ru1—N5—C21—C22	179.9 (3)	C48—C49—C50—C51	178.3 (4)
Ru1—N5—C28—N6	0.1 (6)	C49—C50—C51—C52	−0.3 (8)
Ru1—N5—C28—C24	−179.8 (3)	C50—C51—C52—C53	−178.0 (5)
C21—N5—C28—N6	177.7 (4)	C50—C51—C52—C56	3.1 (7)
C21—N5—C28—C24	−2.2 (6)	C51—C52—C53—C54	−179.0 (5)
C28—N5—C21—C20	−179.2 (4)	C51—C52—C56—N11	−2.9 (7)
C28—N5—C21—C22	2.0 (7)	C51—C52—C56—N12	179.2 (4)
C27—N6—C28—N5	179.4 (4)	C53—C52—C56—N11	178.1 (4)
C27—N6—C28—C24	−0.7 (7)	C53—C52—C56—N12	0.2 (7)
C28—N6—C27—C26	1.2 (8)	C56—C52—C53—C54	−0.1 (7)
Ru2—N7—C29—C30	−178.4 (4)	C52—C53—C54—C55	1.1 (8)
Ru2—N7—C33—C32	178.9 (4)	C53—C54—C55—N12	−2.4 (9)

### Hydrogen-bond geometry (Å, °)

**Table:**

*D*—H···*A*	*D*—H	H···*A*	*D*···*A*	*D*—H···*A*
O1—H1A···N6	0.81 (3)	1.91 (3)	2.647 (5)	151 (4)
O1—H1B···O4	0.81 (4)	1.98 (4)	2.773 (6)	167 (5)
O2—H2A···N12	0.80 (3)	1.91 (3)	2.637 (5)	151 (4)
O2—H2B···O5	0.81 (4)	1.98 (4)	2.762 (6)	161 (4)
